# 

**Published:** 1851-03

**Authors:** 


The length of the article on the *Rochester knockings* excludes other editorial matter prepared for this No. The reader will observe that the publishers have added two pages to the present No. Several communications received too late for the present No. will appear in our next.

